# 2D and 3D Stem Cell Models of Primate Cortical Development Identify Species-Specific Differences in Progenitor Behavior Contributing to Brain Size

**DOI:** 10.1016/j.stem.2016.03.003

**Published:** 2016-04-07

**Authors:** Tomoki Otani, Maria C. Marchetto, Fred H. Gage, Benjamin D. Simons, Frederick J. Livesey

**Affiliations:** 1 Gurdon Institute and Department of Biochemistry, University of Cambridge, Tennis Court Road, Cambridge CB2 1QN, UK; 2 Laboratory of Genetics, Salk Institute, North Torrey Pines Road, La Jolla, CA 92037, USA; 3 Gurdon Institute, Cambridge Stem Cell Institute, and Department of Physics, University of Cambridge, Tennis Court Road, Cambridge CB2 1QN, UK

## Abstract

Variation in cerebral cortex size and complexity is thought to contribute to differences in cognitive ability between humans and other animals. Here we compare cortical progenitor cell output in humans and three nonhuman primates using directed differentiation of pluripotent stem cells (PSCs) in adherent two-dimensional (2D) and organoid three-dimensional (3D) culture systems. Clonal lineage analysis showed that primate cortical progenitors proliferate for a protracted period of time, during which they generate early-born neurons, in contrast to rodents, where this expansion phase largely ceases before neurogenesis begins. The extent of this additional cortical progenitor expansion differs among primates, leading to differences in the number of neurons generated by each progenitor cell. We found that this mechanism for controlling cortical size is regulated cell autonomously in culture, suggesting that primate cerebral cortex size is regulated at least in part at the level of individual cortical progenitor cell clonal output.

## Highlights

• Human and primate PSCs can replicate cortical development in culture
• PSC-derived cortical progenitors from different species expand to different degrees
• Clonal analysis reveals marked difference in neurogenesis output over time
• Species-specific timing differences in neurogenesis are regulated cell autonomously

## Graphical Abstract

**Figure:**
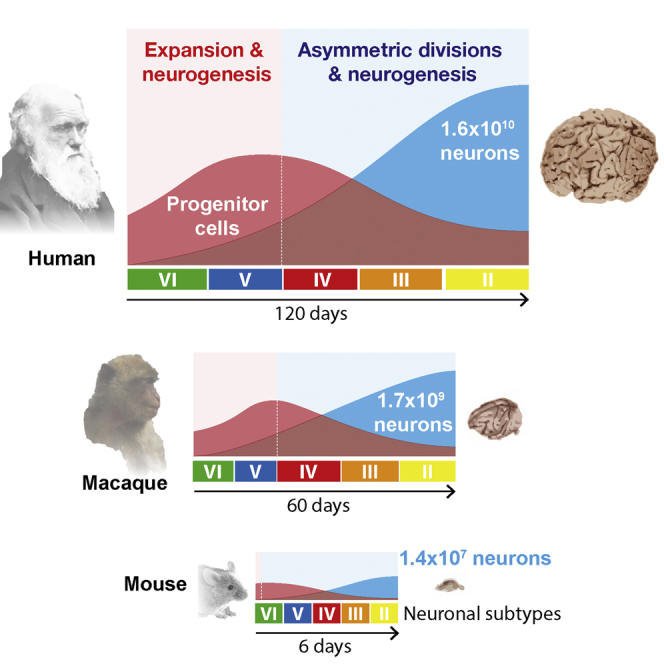

## Teaser

Based on modeling of cortical neurogenesis with pluripotent cells in 2D and organoid systems, Otani et al. suggest that species-specific differences in cortical size and cognitive ability between human and other animals result at least in part from cell-autonomous differences in cortical progenitor proliferation before neurogenic differentiation.

## Introduction

The cerebral cortex is the integrative and executive center of the mammalian CNS, making up over three-quarters of the human brain (Mountcastle et al., 1998). An increase in neuronal number, and thus cerebral cortex size, is thought to provide a template for more complex neural architectures, contributing to differences in cognitive abilities between humans and other primates (Geschwind and Rakic, 2013, Herculano-Houzel, 2012). The developmental mechanisms that generate differences in neuronal number and diversity, and thus cerebral cortex size in humans, other primates, and mammals in general, are currently poorly understood.

During embryonic development, all excitatory cortical projection neurons are generated directly or indirectly from neuroepithelial progenitor cells of the cortical ventricular zone (VZ) (Rakic, 2000). A common feature of cerebral cortex development in all mammals is that multipotent cortical progenitor cells produce multicellular clones of neurons over developmental time, generating different classes of cortical projection neurons and then glial cells in fixed temporal order (Kornack and Rakic, 1995, McConnell, 1988, McConnell, 1992, Walsh and Cepko, 1988). Neuroepithelial cells are the founder progenitor cell population in the cerebral cortex, giving rise to neurogenic radial glial cells (RGCs) that generate all of the excitatory neurons of the cerebral cortex, either directly or indirectly (Florio and Huttner, 2014, Mountcastle et al., 1998). RGCs can self-renew (proliferate), directly generate postmitotic neurons, or produce two different types of neurogenic progenitor cells: intermediate/basal progenitor cells (IPCs) and outer RGCs (oRGCs) (Florio and Huttner, 2014, Geschwind and Rakic, 2013, Herculano-Houzel, 2012, LaMonica et al., 2012). Both basal progenitor cells and oRGCs can also self-renew or generate neurons, with some evidence that IPCs have limited proliferative capacity (Gertz et al., 2014, Rakic, 2000).

Although several different processes have been proposed to contribute to increased neuronal numbers in the primate cortex (Herculano-Houzel, 2009), research has focused on two primary mechanisms: an increase in the number of founder neuroepithelial cells, driven by increased proliferation of neuroepithelial cells before entering the neurogenic period of cortical development (Florio and Huttner, 2014, Geschwind and Rakic, 2013), and an increase in the number of oRGCs, as found in primates (Hansen et al., 2010). The latter in turn amplify the output of RGCs (for a recent review, see Dehay et al., 2015). The radial unit hypothesis proposes that an increase in the number of founder neuroepithelial cells is the basis for the increase in cortical size in humans compared with other primates (Geschwind and Rakic, 2013, Rakic, 2000). The identification of oRGCs in primates and other mammals has led to a modification of the radial unit hypothesis to suggest that the addition of oRGCs effectively increases the progenitor population and thus is a major contributor to primate cortical expansion (Fietz et al., 2010, Hansen et al., 2010, Smart et al., 2002).

Current models for the cellular mechanisms that generate the increased numbers of neurons found in the primate cerebral cortex rely on extrapolating from a large body of work on rodent, primarily mouse, cortical neurogenesis. However, the cortex of humans and other primates appears to follow different scaling rules than that of other mammals, including mouse, in terms of the relationship between cortical volume and cell number and overall body size (Azevedo et al., 2009). We and others have developed human stem cell systems to study cerebral cortex neurogenesis in vitro (Espuny-Camacho et al., 2013, Mariani et al., 2012, Shi et al., 2012a), finding that directed differentiation of human pluripotent stem cells (PSCs) to cerebral cortex progenitor cells robustly replays the temporal order of cortical neurogenesis, including the production of the diversity of progenitor cell types found in vivo (Shi et al., 2012a).

In this study, we extended the use of stem cell systems to compare human, macaque, and chimpanzee cortical neurogenesis to understand the developmental mechanisms regulating increased cortical size in different primates. We find that there are several important differences in cerebral cortex progenitor cell biology between rodents and primates, and between humans and nonhuman primates, that contribute to the marked differences in neuronal number among the different species. Together, these findings constitute multiple new insights into the biology of generating large brains in relatively slowly developing mammals, including primates.

## Results

### Replication of Species-Appropriate Developmental Timing of Cortical Neurogenesis In Vitro from PSCs of Multiple Primate Species

We used stem cell systems to analyze the relationships between progenitor cell proliferation dynamics, clonal output, neuronal number, and cortical size in four species of primate with differing brain sizes. We compared cortical neurogenesis among humans; the chimpanzee *Pan troglodytes* (Marchetto et al., 2013), a great ape with less than half the number of cortical neurons of humans (Herculano-Houzel et al., 2007); and in two species of Old World monkey, the crab-eating macaque, *Macaca fascicularis*, and the southern pig-tailed macaque, *Macaca nemestrina*, both of which have cerebral cortices with approximately one-tenth the numbers of neurons as humans (Herculano-Houzel et al., 2007).

**Figure 1:**
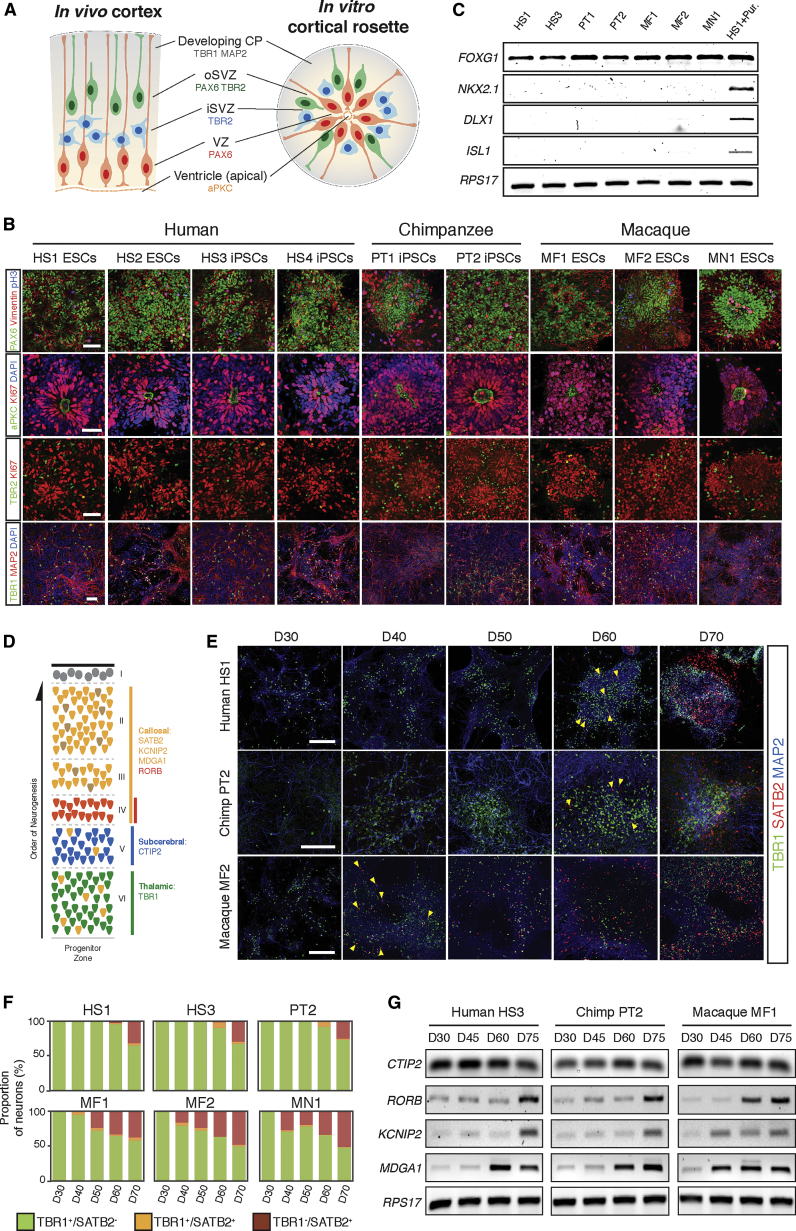
Replication of Species-Appropriate Developmental Timing of Cortical Neurogenesis In Vitro from PSCs of Multiple Primate Species (A) Schematic comparing the in vivo developing cortical neuroepithelium and in vitro, stem-cell-derived cortical neuroepithelial rosette. In a cortical rosette, the aPKC^+^ apical surface is at the rosette center, immediately surrounded by the VZ-like region containing PAX6/Vimentin^+^ RGPCs. Outside the VZ, there is no clear positional distinction between inner subventricular zone (iSVZ) and outer SVZ (oSVZ), where both TBR2^+^ intermediate progenitor cells (IPCs) and PAX6/Vimentin^+^ outer RGP-like cells are found. These progenitor cells produce cortical neurons (such as TBR1/MAP2^+^ thalamic projection neurons), which then migrate away from the rosette center. (B) Representative immunofluorescence images of cortical neuroepithelial rosettes derived from human (HS1, HS2, HS3, and HS4), chimpanzee (PT1 and PT2), and macaque (MF1, MF2, and MN1) PSCs. Antibodies used are as indicated: PAX6/Vimentin (RGPCs), aPKC (apical cell domain), TBR2/Ki67 (IPCs), or TBR1/MAP2 (layer VI cortical neurons). Scale bars, 50 μm. (C) Semiquantitative RT-PCR for the cortically expressed transcription factor (TF), *FOXG1*, and ventrally/caudally expressed TFs, *NKX2.1*, *DLX1*, and *ISL1*. All cortical progenitor cells from human and nonhuman primate PSCs are dorsal pallial in regional identity, unless treated with the Smoothened/Hedgehog agonist purmorphamine (HS1 + Pur.) during induction to ventralize the progenitor cells to noncortical identities. (D) The cerebral cortex is organized into six layers of excitatory projection neurons with defined gene-expression profiles, based on detailed studies of the mouse cerebral cortex (Molyneaux et al., 2007): thalamic projection neurons in layer VI express TBR1, subcerebral projection neurons in layer V express CTIP2, and callosal projection neurons in layer II–IV express RORB, SATB2, KCNIP2, and MDGA1. (E) Immunofluorescence images of in vitro-derived cortical neurons of human, chimpanzee, and macaque at the indicated developmental stages post-cortical induction (days 30–70). Cultures were stained for TBR1 and SATB2 to monitor differentiation of deep- and upper-layer neurons (yellow arrowheads indicate first SATB2^+^ neurons generated). Scale bars, 200 μm. (F) Quantification of the relative proportions of TBR1^+^ and SATB2^+^ neurons in human (HS1 and HS2), chimpanzee (PT2), and macaque (MF1, MF2, and MN1) cultures at the indicated developmental stages (days 30–70). (G) Semiquantitative RT-PCR of expression of *CTIP2* (layer V), *RORB* (layer IV), *KCNIP2*, and *MDGA1* (layer II–IV) at the indicated stages in human, chimpanzee, and macaque cortical cultures. Transcripts enriched in later-born, upper-layer neurons (*RORB*, *KCNIP2*, and *MGDA1*) are expressed at an earlier stage in macaque than in humans or chimpanzee.

We applied our previously described methods for directed differentiation of human PSCs to cerebral cortex to generate cortical progenitor cells of each species (Shi et al., 2012a, Shi et al., 2012b). Following neural induction, neuroepithelial cells generated the different populations of progenitor cells found in the mammalian cerebral cortex, including RGCs and IPCs (Figures 1A and 1B). These progenitor cells were arranged in characteristic rosette structures, composed of polarized RGCs with their apical surfaces concentrated at the rosette center, and IPCs located at the basal/peripheral region of the rosette (Shi et al., 2012a). Neuroepithelial rosettes were confirmed as dorsal pallial in regional identity by positive and negative expression of region-specific transcription factors (Figure 1C).

To further investigate whether in vitro directed differentiation accurately captured in vivo progenitor cell diversity, we labeled individual cortical progenitor cells by lentiviral infection with GFP expression constructs to observe progenitor cell morphologies and cell division types. Bipolar progenitor cells, with characteristic ventricular RGC (vRG) morphology, were found within rosettes, whereas unipolar progenitor cells, with typical oRGC morphologies, were found at the periphery of rosettes (Figure S1A, available online). Live imaging demonstrated that the different progenitor cell types underwent characteristic, cell-type-specific mitotic cell-body movements (Gertz et al., 2014, Ostrem et al., 2014): vRGs displayed interkinetic nuclear migration, whereas oRGCs underwent mitotic somal translocation (Figures S1B and S1C).

Excitatory glutamatergic neurons destined for each cortical layer are produced in a fixed temporal order during development, beginning with layer VI (TBR1^+^) neurons, followed by neurons of each of the other five layers (Figure 1D) (Mountcastle et al., 1998). The fixed order of cortical neuron production was preserved in vitro for all nonhuman primates (Figure 1E), as we previously reported for humans (Shi et al., 2012a). Furthermore, the timing of generation of different cell types followed species-specific timing in vitro. All species generated layer VI neurons at approximately the same stage in vitro (20 days after initiating neural induction from PSCs), as observed in vivo (Workman et al., 2013).

Both human and chimpanzee cortical progenitor cells switched from deep to upper layer neurogenesis 40–50 days later, as indicated by the appearance of SATB2^+^ layer II–IV neurons (Figures 1E and 1F). This finding is consistent with the approximately 45-day interval between layer VI and layer IV genesis in the developing human embryo (Workman et al., 2013). In contrast, cortical progenitor cells from both macaque species switched to upper-layer neuron production less than 20 days after deep-layer neurogenesis, reflecting the reported 19-day interval between these developmental events in vivo (Workman et al., 2013). The difference in the timing of the differentiation of upper-layer neurons between human, chimpanzee, and macaques was further confirmed by analyzing the time course of expression of additional genes specifically expressed by neurons of layers II–IV and V and VI (*CTIP2*, layer V and VI; *RORB*, *KCNIP2*, and *MDGA1* for layers II–IV; Figure 1G).

### Timing of Cortical Neurogenesis Is Independent of Neuronal Lamination and 3D Organization

**Figure 2:**
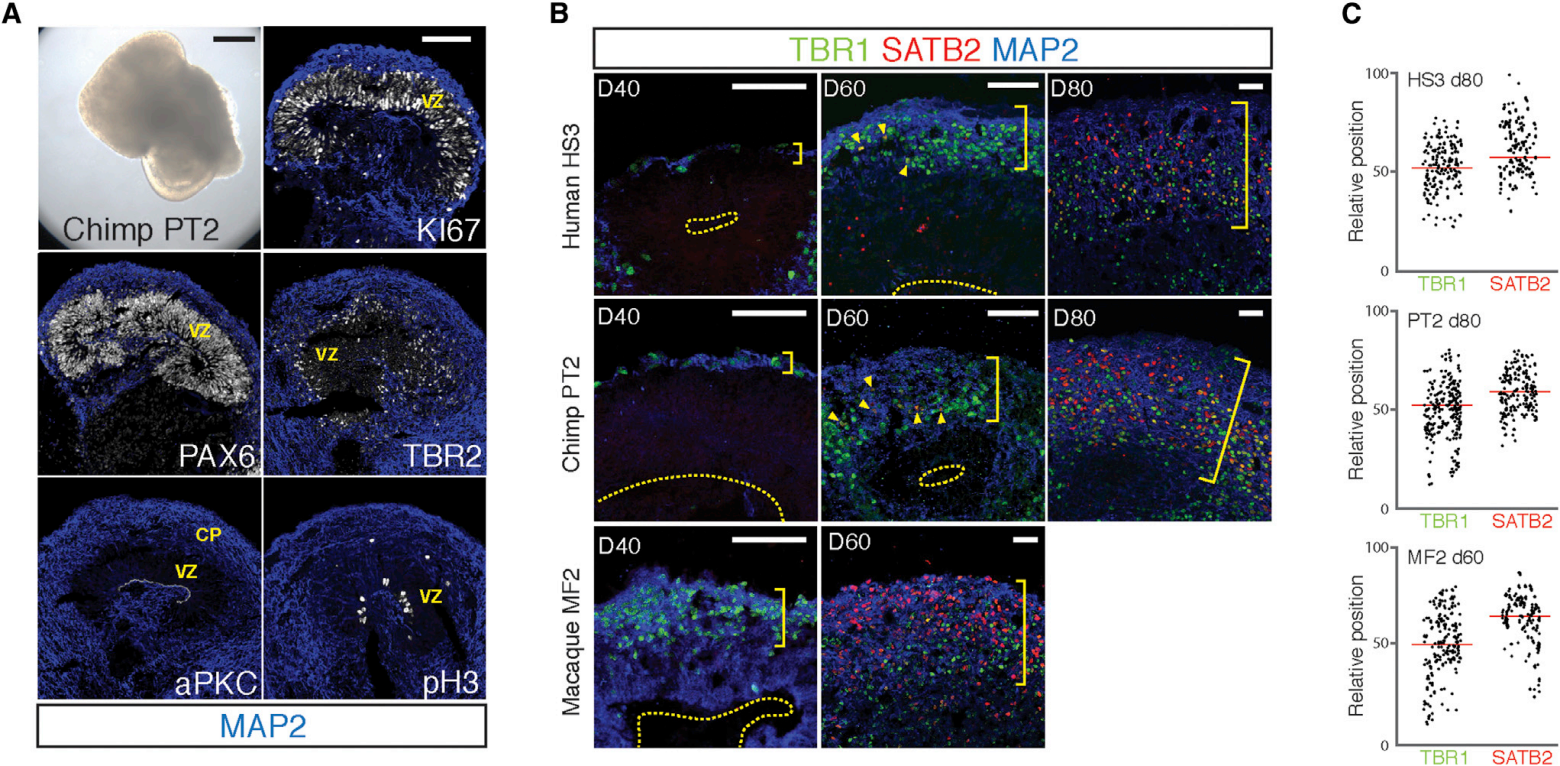
Timing of Cortical Neurogenesis Is Independent of Neuronal Lamination and 3D Organization (A) Chimpanzee cerebral cortex organoids (scale bar, 200 μm). Organoids develop in vivo-like organization of VZ, with PAX6^+^/Ki67^+^ polarized (apical aPKC localization) progenitor cells within the VZ, apical mitoses (pH3^+^ cells), and IPCs at the outer margin of the VZ. Antibodies as indicated in each panel. Scale bar, 100 μm (B) Human, chimpanzee, and macaque cortical organoids undergo sequential production of TBR1^+^ deep-layer neurons and SATB2^+^ upper-layer neurons (yellow arrowheads indicate initial SATB2^+^ neurons generated). As organoids developed for longer periods, cortical neurons migrated to form cortical plate-like structures (yellow bracket) with some separation of layers of TBR1^+^ and SATB2^+^ neurons. Scale bars, 50 μm. (C) Scatterplots of positions of TBR1^+^ and SATB2^+^ neurons relative to the ventricular surface in human day 80, chimpanzee day 80, and macaque day 60 cortical organoids. Red lines represent median positions.

We observed conservation of development timing of cortical neurogenesis using differentiation of adherent, polarized neuroepithelial rosettes. Under these culture conditions, cortical neurons are highly migratory and form dense cultures that are 100–200 μm thick (Kirwan et al., 2015). However, they do not form the ordered layers of projection neurons (laminae) found in the cortex in vivo (Kirwan et al., 2015). To investigate whether lamination altered development timing, we also studied the timing of differentiation of deep- and upper-layer cortical neurons in nonadherent, three-dimensional (3D) cortical organoids that underwent some degree of lamination and resembled the in vivo cortex in terms of the spatial relationships of the progenitor cell populations and the postmigratory neurons (Figure 2A) (Kadoshima et al., 2013).

As in the nonlaminating rosette system, we found that deep-layer TBR1^+^ cortical neurons appeared first in each species (Figure 2B), followed by SATB2^+^ upper-layer neurons that migrated to the basal/outer surface (Figures 2B and 2C). The timing of the interval between the appearance of deep- and upper-layer neurons in organoids was in line with that which we observed in the rosette system for humans, chimpanzees, and macaques. Upper-layer neurons were present in large numbers in macaque cortical organoids at day 60, at which stage there were few upper-layer, SATB2^+^ neurons in the human and chimpanzee organoids (Figure 1B). At day 80 in human and chimpanzee organoids, there was a substantial population of SATB2^+^/TBR1^−^ upper-layer neurons that had migrated and began laminating near the outer/pial surface (Figures 2B and 2C).

### Functional Maturation of Primate Cortical Neurons Demonstrates Species-Specific Timing

**Figure 3:**
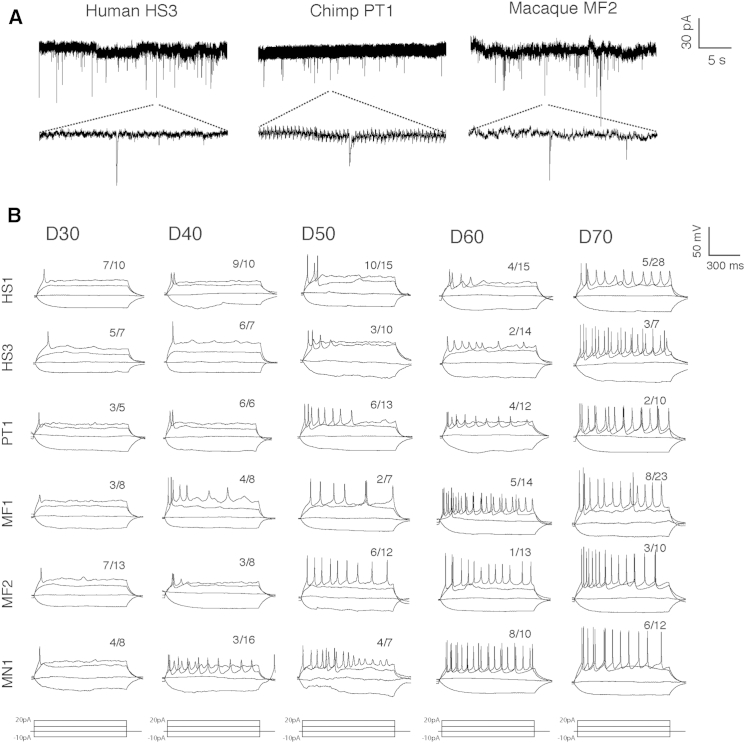
Functional Maturation of Primate Neurons Demonstrates Species-Specific Timing (A) Detection of miniature excitatory postsynaptic currents (mEPSCs) in whole-cell recordings of human (HS2), chimpanzee (PT1), and macaque (MF2) cortical neurons. Spontaneous depolarizations indicate the presence of synaptic activity. (B) Patch-clamp, single-neuron recordings of electrophysiological properties of cortical neurons at different developmental stages (days 30–70) for human (HS1 and HS2), chimpanzee (PT1), and macaque (MF1, MF2, and MN1). In response to stepwise current stimulation (−10 to 20 pA), in vitro cortical neurons fired action potentials (APs). The response to current injection evolved over time, with mature neurons firing more APs following single stimuli. Numbers represent frequencies of patterns of AP firing at each given developmental stage.

We previously found that in vitro-derived human cortical neurons undergo electrophysiological maturation over a prolonged period, compared with rodents, as also occurs in vivo (Shi et al., 2012a). To investigate the developmental maturation of nonhuman primate cortical neurons, we performed single-neuron patch-clamp recordings of human, chimpanzee, and macaque neurons. Miniature excitatory postsynaptic currents were detected in neurons of each primate species, confirming that neurons in each case efficiently formed functional synapses (Figure 3A).

Using action potential firing in response to current injection as a measure of neuronal maturity, we found that macaque neurons of both species matured more quickly than both human and chimpanzee (Figure 3B). Analyzing neuronal maturity at a range of developmental stages (days 30–70), we found that functionally mature neurons were present at an earlier stage and at higher frequency in macaques than in humans and chimpanzees (Figure 3B). Therefore, consistent with differential developmental timings of neurogenesis for each primate species, the maturation of cortical neurons also reflected species-specific timing in vitro, with humans and chimpanzees demonstrating similar rates of neuronal maturation.

### Clonal Analysis Reveals Marked Differences between Human and Macaque Cortical Progenitor Cell Dynamics over Developmental Time

**Figure 4:**
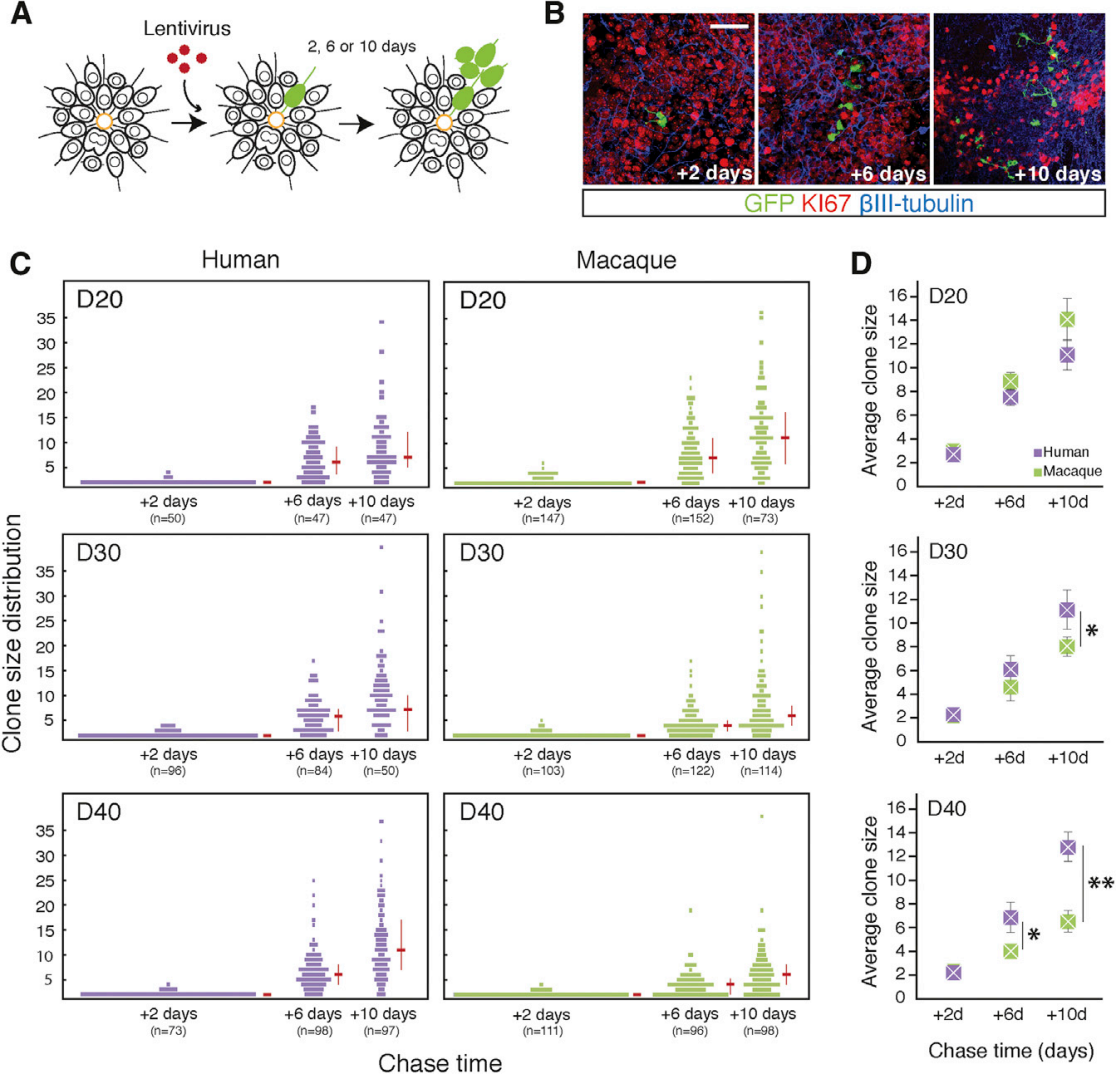
Clonal Analysis Reveals Marked Differences between Human and Macaque Cortical Progenitor Cell Dynamics over Developmental Time (A) Single cortical progenitor cells were labeled with low-titer, replication-incompetent lentiviruses at clonal resolution (see Supplemental Experimental Procedures for further details). Following infection at days 20, 30, or 40, progenitor cells were cultured for 2, 6, or 10 days, fixed, and immunostained for analysis. (B) Representative immunofluorescence images of clones derived from a single progenitor cell after 2, 6, or 10 day chase periods (panels as labeled) and immunostained for Ki67 (cycling progenitor cells) and βIII-tubulin (postmitotic neurons). Scale bar, 100 μm. (C) Human and macaque clone size distributions for each developmental stage (days 20, 30, and 40) at each time point postlabeling (2, 6, and 10 days). Red horizontal bars represent medians, and vertical bars indicate the interval between the first and third quartiles of the clonal distribution. Data for each species are combined from four human pluripotent cell lines (two ESCs and two iPSCs) and from three macaque ESC lines. Total number of clones analyzed for each line is as follows: HS1, n = 440; HS2, n = 43; HS3, n = 201; HS4, n = 93; MF1, n = 247; MF2, n = 469; MN1, n = 303. (D) Human and macaque average clone sizes for time points shown in (C). Significant differences between the average sizes of human and macaque clones at day 30 + 10 (p = 0.0437), day 40 + 6 (p = 0.0154), and day 40 + 10 (p = 0.205 × 10^−2^) are labeled. Error bars, SD.

The number of neurons generated by a cortical progenitor cell (clone size) is a major contributor to total cell number and thus overall size of the cerebral cortex. Clonal lineage analysis of in vitro-derived cortical progenitor cells enables detailed comparisons of cortical progenitor cell dynamics and clonal outputs between species. Given the marked differences in cortex size, cortical neuronal number, and developmental timing between humans and macaques, we focused our analyses on comparing cortical progenitor cell outputs between those species. Single-cell clonal analysis was carried out using GFP-expressing, replication-incompetent lentiviral labeling of individual progenitor cells at 10-day intervals (days 20, 30, or 40 post-cortical induction; Figures 4A and 4B).

Clones (comprised of two cells or more and therefore rooted in labeled progenitor cells) were collected and analyzed 2, 6, and 10 days after labeling, generating data on clone size distributions for progenitor cells labeled at each developmental stage (days 20, 30, and 40). Clonal analyses were carried out in multiple PSC lines in humans (embryonic stem cells [ESCs] and induced PSCs [iPSCs]) and macaque (ESCs). The accuracy of our assignment of clone membership was tested by two methods. First, we tested whether our sparse labeling method, using low-titer viral infection, led to more than one infection event in close proximity. Mixing mCherry and GFP-expressing viruses before infection and clone labeling demonstrated that the occurrence of mixed GFP/mCherry-labeled clones was extremely rare (Figure S2A). Second, using nearest neighbor analysis, we analyzed the spatial distribution of labeled cells and found that it was highly improbable, assuming a starting random distribution of single labeled cells, that clones were merged separate infection events, as a consequence of clonal expansion and/or migration (Supplemental Experimental Procedures; Figures S2B and S2C).

In each cohort of progenitor cells labeled at the different time points (days 20, 30, or 40) in both species, we observed a steady increase in average clone size over the 10-day period after labeling (Figure 4C). For humans, we observed that the increase in overall clone size over the 10 days after labeling was very similar in cortical progenitor cells labeled at each developmental age (Figure 4C). In contrast, clone size distributions in macaques changed between days 20 and 40. The clone size distributions from the macaque day 20 time course were similar to those in humans. However, clones generated by progenitor cells labeled at days 30 and 40 did not expand to the same degree as those at day 20 (Figure 4C).

Importantly, we saw little variation in proliferative behaviors of progenitor cells derived from different cell lines of the same species (Figure S2D). Reflecting the interspecies difference in clonal expansion, we found that the average size of clones at each time point diverged between humans and macaques later in development, with older macaque cortical progenitor cells making significantly smaller clones on average than human progenitor cells (Figure 4D). Therefore, macaque progenitor cells underwent a change in proliferative behavior over the day 20–day 40 period, leading to a reduction in total clone size. In contrast, human progenitor cells did not alter their proliferative behavior and clonal outputs over this time period.

### Differences in Clone Growth between Macaque and Human Are Reflected in Differences in Progenitor Cell Proliferative Behaviors

**Figure 5:**
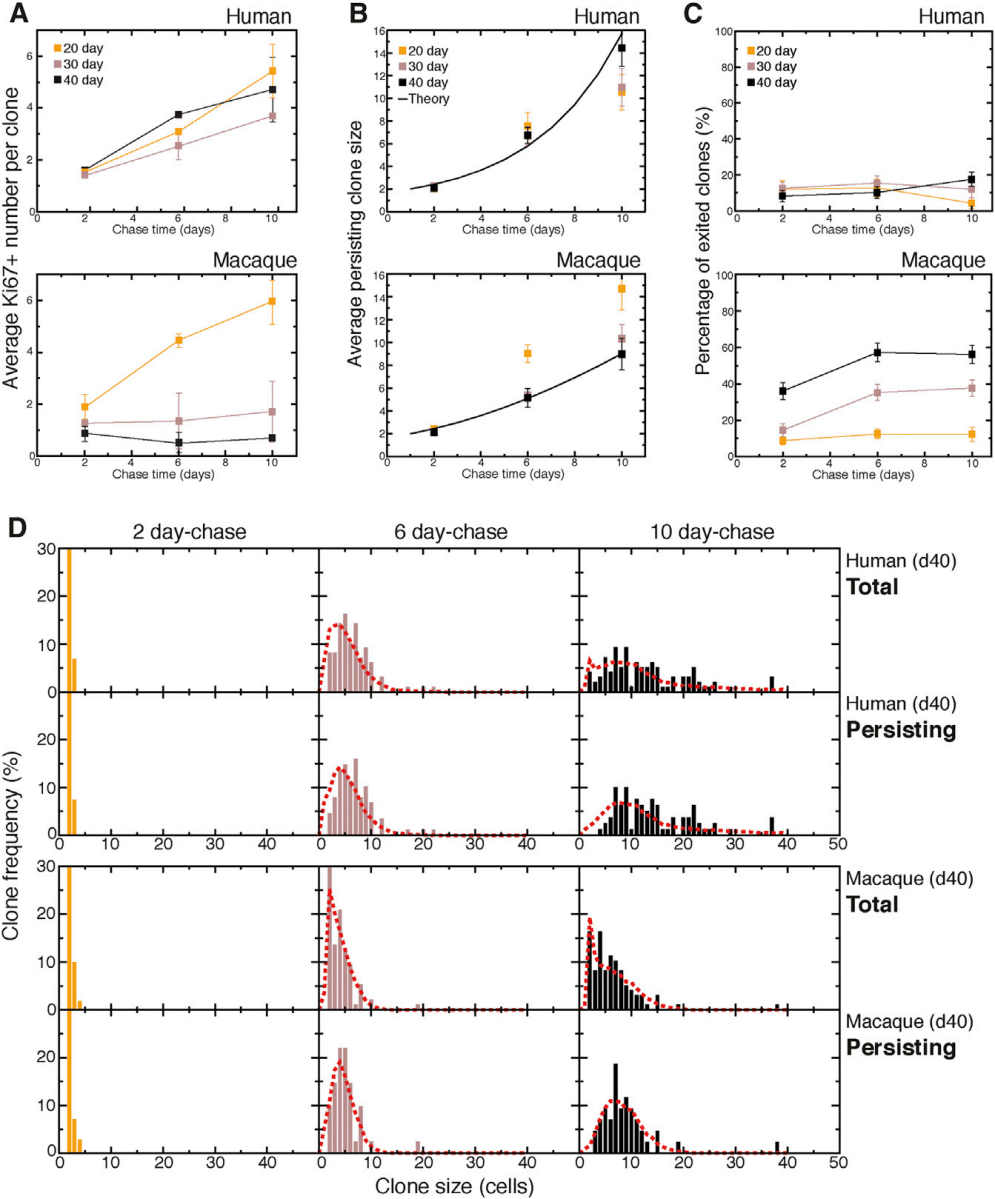
Differences in Clone Growth between Macaque and Human Are Reflected by Differences in Progenitor Cell Proliferative Behaviors (A) Quantification of the average number of Ki67^+^ progenitors in a human or macaque clone after various chase periods (2, 6, and 10 days) following clonal labeling at days 20, 30, and 40. Data analysis for this and subsequent panels is from two human lines (HS1 and HS3) and three macaque lines (MF1, MF2, and MN3). Error bars, SD. (B) Average size of all “persisting” clones (which contain one or more Ki67^+^ progenitor cells) with different chase periods following clonal labeling at days 20, 30, and 40. The black solid line represents the theoretically predicted values for persisting clone expansion following day 40 labeling (see Supplemental Experimental Procedures for further details on the computational model). Error bars, SD. (C) Percentage of human and macaque “exited” clones (which no longer contain any Ki67^+^ progenitor cells) with different chase periods after clonal labeling at days 20, 30, and 40. Error bars, SD. (D) Human and macaque clone size distributions of total and persisting clones after clonal labeling at day 40 and analysis 2, 6, and 10 days after labeling. Red dotted lines represent theoretically predicted values (see Supplemental Experimental Procedures for details of computational modeling).

Clonal lineage data suggest that macaque progenitor cells undergo a time-dependent change in their proliferative behavior, which would reduce the numbers of progenitor cells per clone at later stages of development. We investigated this finding further by analyzing clone composition in terms of neurons and progenitor cell numbers. We found that the average number of progenitor cells per clone (as assessed by Ki67 expression) increased over the 10 days after labeling at all developmental stages in humans (Figure 5A). In macaques, the number of progenitor cells plateaued at an average of around just one progenitor cell per clone at later stages of development (Figure 5A), consistent with the reduction in clonal output by later-stage macaque progenitor cells. This finding suggested that human and macaque progenitor cells had distinct proliferative behaviors at later developmental ages, with human progenitor cells continuing to expand their population for a longer period than macaque.

The observed changes in proliferative behavior ought to be underpinned by differences in progenitor cell division types. To gain further insight into the division patterns of progenitor cells, we judged each clone as either persisting or exited, depending on whether the clone contained at least one progenitor cell (Figures 5B and 5C). Analysis of the size distribution of persisting clones, representing the population of clones containing at least one Ki67^+^ cell, revealed an approximately exponential increase in average clone size for human progenitor cells labeled at day 40, compared to a linear-like increase in macaque (Figure 5B). This finding suggested that a higher proportion of human progenitor cells were dividing symmetrically to generate additional progenitor cells, whereas macaque progenitor cells followed a more asymmetric or neurogenic division pattern. We found that approximately 15% (human) and 60% (macaque) of progenitor cells had exited proliferation 10 days after labeling (Figure 5C), implying that the majority of macaque progenitors were terminally differentiating at this stage.

Clonal analysis suggests that macaque progenitor cells cease their progenitor expansion phase earlier in development than human. To test this, we applied a computational model informed by the findings of a recent in vivo genetic labeling study of cortical neurogenesis in mouse that showed that cortical progenitor cells transit sequentially through a symmetrical proliferative phase to a neurogenic phase in which cells make a sequence of asymmetric cell divisions giving rise to IPCs, the latter having variable but limited proliferative potential (Gao et al., 2014).

Using experimentally measured parameters of apoptosis (Figures S3A and S3B) and cell-cycle length (Figure S4), we found that such hypotheses, and hence the model (Supplemental Experimental Procedures), could explain the differences in clonal behaviors between human and macaque, including the distribution of clone size and composition, as well as the frequency of terminally differentiated clones at the latest (10 day) time point (Figures 5D, S3C, and S3D). Therefore, the clonal analysis data and the computational model together demonstrated that human cortical progenitor cells had an extended period (days 20–50) during which production of cortical neurons was balanced with production of additional progenitor cells. This finding is in contrast with macaque progenitor cells, which switched much earlier (at around day 35) to a more neurogenic program at the expense of production of progenitor cells.

### Testing Predictions of Progenitor Cell Proliferative Behaviors during Human and Macaque Cortical Development

**Figure 6:**
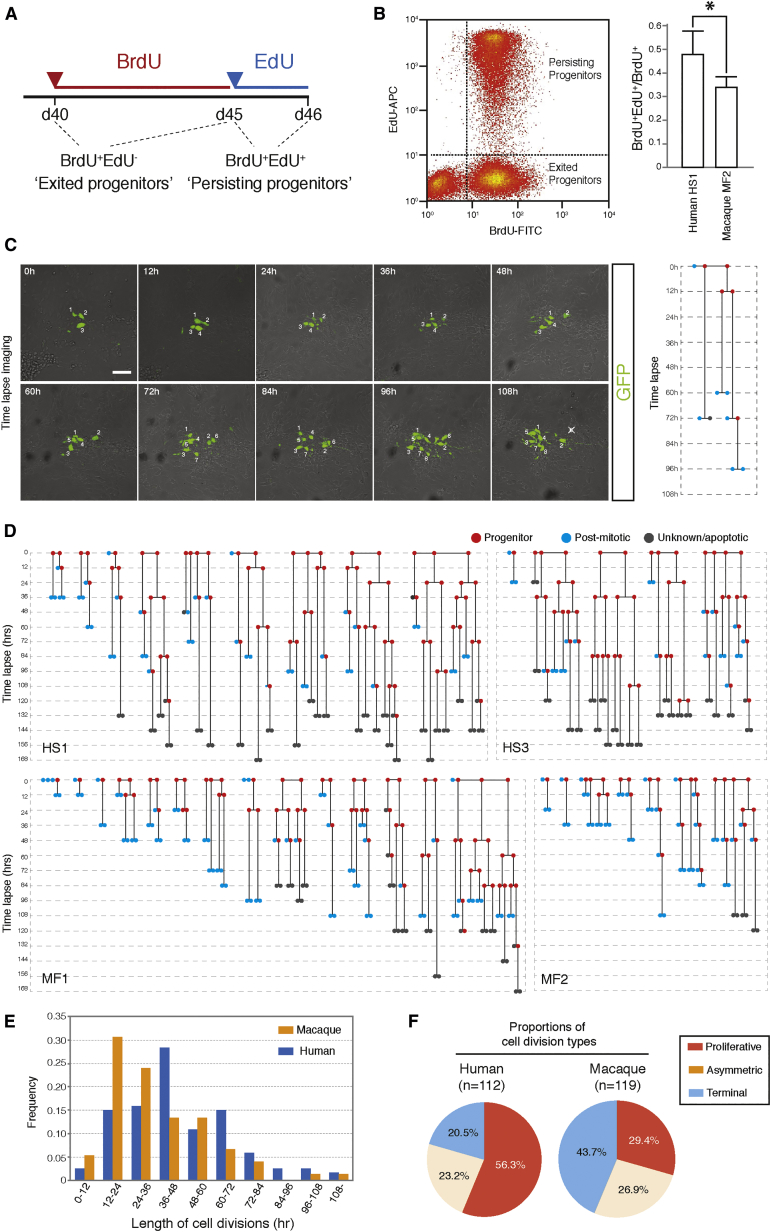
Testing Predictions of Progenitor Cell Proliferative Behaviors during Human and Macaque Cortical Development (A) Experimental design of BrdU/EdU double-labeling assay. From day 40, BrdU was added to human and macaque cortical cultures to cumulatively label all progenitor cells and their progeny until day 45, at which point BrdU was switched to EdU to reveal the ratio of persisting progenitor cells (BrdU^+^EdU^+^) to exited progenitor cells (BrdU^+^EdU^−^). (B) Representative scatterplot of EdU/BrdU double-labeling assay analyzed by flow cytometry. Three distinct populations of cells are evident: BrdU^−^EdU^−^ noncycling cells (which were postmitotic at the beginning of the experiment), BrdU^+^EdU^−^ exited progenitor cells, and BrdU^+^EdU^+^ persisting progenitor cells. The proportion of progenitor cells persisting after 5 day chase (BrdU^+^EdU^+^/all BrdU^+^) is higher for human than macaque (p = 0.0119). Human data average of n = 4; macaque data average of n = 5. Error bars, SD. (C) Time-lapse imaging of human and macaque cortical progenitor cell divisions. GFP-labeled progenitors in clones were followed and imaged every 12 hr over a period of 168 hr. From the sequential images, a lineage tree of clonal progenitor divisions was reconstructed. Cells were assigned as “progenitor” if they divided in the span of recording (red circle), “postmitotic” if they did not divide for more than 60 hr (equivalent to the third quartile of the distribution of all cell divisions recorded) (blue circle), or “unknown/apoptotic” if cells either disappeared from the imaging frame or were born close to the end of filming period (gray circle). (D) Representative lineage trees showing cell divisions of human (HS1 and HS2) and macaque (MF1 and MF2) progenitors, reconstructed from sequential images. (E) Bar graphs showing distributions of the lengths of cell cycles based on reconstructed lineage trees for human (blue) and macaque progenitor cells (orange). (F) Pie charts showing proportions of cell division types for human and macaque progenitors, based on reconstructed linage trees. “Proliferative” divisions are those giving rise to two progenitors, “asymmetric” divisions giving one progenitor and one postmitotic cell, and “terminal” division giving two postmitotic cells.

To assess the validity of the model and the findings of the clonal analyses, we carried out two different experiments to analyze the proliferative capacity and division types of human and macaque progenitor cells. First, we designed a strategy to assess the proliferative capacity of human and macaque cortical progenitor cells between days 40 and 46. This is the critical time window that we identified during which human and macaque progenitor cell division types diverge in their proliferative potential. We made use of an EdU/BrdU double-labeling strategy to first label all cycling progenitor cells and their progeny over a 5-day interval with BrdU, followed by a final 24 hr EdU pulse to identify the fraction of that population that was still cycling (i.e., were cycling progenitor cells; Figure 6A).

We found that some 31.2% (macaque) and 48.2% (human) of the progeny of day 40 progenitor cells entered into cell cycle between days 5 and 6 after initial labeling (Figure 6B). These numbers compare favorably with the model that, according to the clonal fits, predicted that some 29% (macaque) and 44% (human) of the progeny would have re-entered into cycle over this time interval (Supplemental Experimental Procedures).

In separate experiments, we used live time-lapse imaging to visualize progenitor cell division types in both species over 7 days (168 hr from day 38 of post-cortical induction) to directly measure the proportion of different progenitor cell division types (Figure 6C). In all, 21 human lineages (from two separate experiments) and 22 macaque lineages (from two separate experiments) were analyzed (Figure 6D). A wide range of cell-cycle lengths was observed in each species, between 12 and over 100 hr, with a mean cell-cycle length in macaque of 36.2 hr compared with 46.5 hr in human (Figure 6E). These averages from direct observations were consistent with the Pax6-positive population cell-cycle length averages measured by cumulative EdU labeling of 47.1 hr in human and 37.7 hr in macaque (Figure S4).

Progenitor cell divisions were designated as proliferative (generating two progenitor cells), neurogenic (generating one progenitor and one neuron), or terminal (generating two neurons), depending on the outcome of the subsequent round of division. Divisions were only defined as neurogenic if one of the two cells did not re-enter cell cycle during the entire imaging period (Figure 6C). The majority of human progenitor cell divisions were proliferative (56.3% of 112 divisions; Figure 6F), compared with 29.4% of macaque divisions (of a total of 119; Figure 6F). Conversely, 43.7% of macaque divisions were terminal (generating two postmitotic cells), compared with 20.5% of human divisions. The frequency of asymmetric divisions was similar in both species: 23.3% in human and 26.9% in macaque (Figure 6F). These measurements of the proportion of cell-division types directly confirm that between days 38 and 45, human progenitor cells are more likely to proliferate or self-renew, whereas macaque progenitor cells undergo neurogenic/terminal pattern of cell divisions.

We conclude that the experimental and theoretical data are consistent with a model for human cortical neurogenesis that proposes an extended period during which progenitor cell expansion is combined with ongoing neurogenesis, reflected in differences in the proliferative behavior of progenitor cells between human and macaque at this stage of development.

### Species-Specific Cortical Progenitor Cell Proliferative Behavior and Developmental Timing Are Regulated by Cell-Autonomous Mechanisms

**Figure 7:**
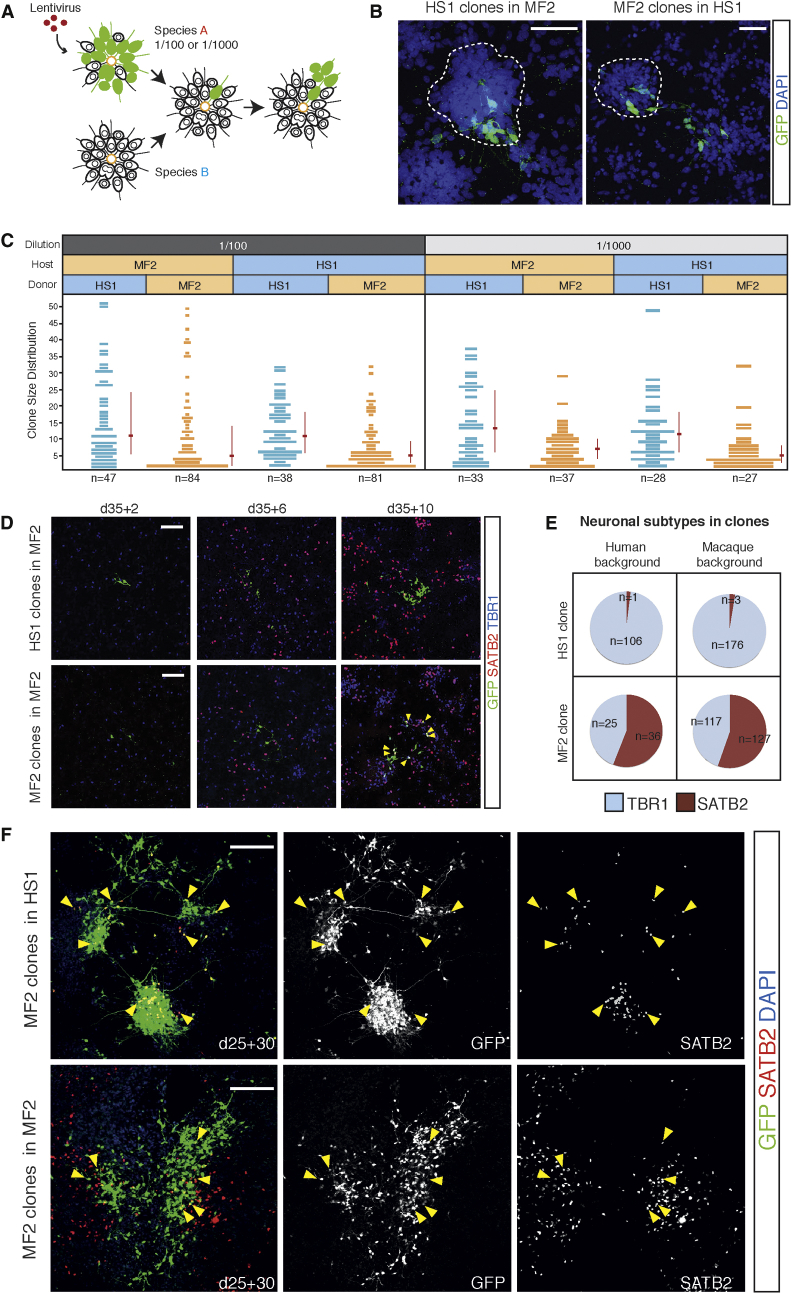
Species-Specific Cortical Progenitor Cell Proliferative Behavior and Developmental Timing Are Regulated by Cell-Autonomous Mechanisms (A) Schematic representation of the experimental design of in vitro, interspecies mixed culture assays. Cortical progenitor cells of species A were labeled with cytoplasmic GFP, delivered by high-titer lentivirus, and subsequently mixed with GFP^−^ progenitors from species B in a 1:100 or 1:1,000 ratio at day 35. Transplanted cortical progenitor cells were cultured with host cells for 2, 6, and 10 days (day 35 + 2, day 35 + 6, and day 35 + 10) before being fixed and immunostained. (B) Immunofluorescence images of GFP^+^ human and macaque clones introduced into macaque and human backgrounds, respectively. GFP^+^ cortical progenitor cells were efficiently incorporated into rosettes of host species (white dotted line). Scale bars, 50 μm. (C) The size distributions of human HS1 clones and macaque MF2 clones in either human or macaque backgrounds at day 35 + 10. Red horizontal lines indicate median clone sizes, and vertical lines show the span between 25% and 75% quartiles for each distribution. n = number of clones analyzed for each culture condition. Dilution of donor cells to host cells (1/100, 1/1,000) is as shown. (D) Representative immunofluorescence images of GFP^+^ human (HS1) or macaque (MF2) clones introduced into macaque background. Cultures were immunostained for transcription factors expressed by deep (TBR1) and upper (SATB2) cortical neurons. Yellow arrowheads indicate SATB2^+^ upper-layer neurons produced from a transplanted macaque progenitor cell. Scale bars, 100 μm. (E) Proportions of TBR1^+^ and SATB2^+^ cortical neurons generated by transplanted progenitor cells of each species in each background as indicated. Host/recipient environment does not affect cell types generated by each species. n = number of cells expressing each transcription factor. (F) Representative immunofluorescence images showing a long-term chimeric mixture of human (HS1) and macaque (MF2) neural progenitors. Single macaque progenitors were introduced into a human (HS1) or macaque (MF2) background at day 25. The mixed cultures were incubated further for 30 days and fixed and stained for the presence of upper-layer cortical neurons (SATB2^+^, yellow arrowheads). Scale bars, 150 μm.

Having established that in vitro-derived cortical progenitor cells demonstrated species-specific cortical progenitor cell clonal behavior and clone-size outputs, we tested whether these features of cortical development were cell autonomous or regulated by cell-cell communication. We performed in vitro, mixed progenitor cell culture assays between and within species, using single GFP-labeled, day 35 human and macaque cortical progenitor cells (Figure 7A). Mixing GFP-labeled progenitor cells at a 1:100 dilution with their host species, we observed that donor progenitor cells were incorporated into host rosettes readily, indicating that the mechanics of cell adhesion and polarity cues were sufficiently similar to enable efficient coculture (Figure 7B).

Transferred progenitor cells proliferated and differentiated to form clones of daughter cells over the subsequent 10-day period (Figure 7C). Analyzing clone size distributions 10 days after setting up mixed cultures, we observed that macaque cortical progenitor cells produced a distribution of clones that tended toward smaller sizes, in which the majority of clones were between two and five cells in size (Figure 7C). This was similar to the distribution of clone sizes measured by lentiviral labeling of day 40 cultures reported above (Figure 4). Importantly, the distribution of clone sizes did not differ when GFP-labeled macaque progenitor cells were mixed with unlabeled macaque progenitor cells or mixed with human progenitor cells (Figure 7C).

The same result was obtained when culturing GFP-labeled human cortical progenitor cells in human or macaque progenitor cell environments (Figure 7C). Human progenitor cells again demonstrated a species-specific distribution pattern of clone sizes, with a wider distribution of clone sizes compared with macaque. Again, this finding was similar to the clone size distribution measured by clonal labeling of day 40 cultures reported above (Figure 4). Notably, the wide distribution of clone sizes was unaffected by the species environment, with similar size distributions observed when placed in macaque or human environments (Figure 7C).

To further explore the contribution of extracellular signaling between progenitor cells, we carried out additional mixing experiments at a lower density of donor cells, culturing GFP-labeled progenitor cells at a dilution of 1:1,000 with unlabeled host cells (Figure 7C). As with the 1:100 experiments, the species environment had no effect on progenitor cell clonal outputs (Figure 7C). Therefore, we conclude that cortical progenitor cell proliferation, differentiation, and clonal outputs are largely regulated cell autonomously in each species.

During the day 35 to day 45 time window, macaque cortical progenitor cells switch from TBR1^+^ deep-layer neurogenesis to the production of SATB2^+^ upper-layer neurons (Figure 7D). We used the mixed species culture system (1:100 dilution) to investigate whether species’ environments could regulate lineage progression, independent of effects on progenitor cell proliferative behaviors.

Clones generated from macaque progenitor cells placed in a macaque background (i.e., macaque to macaque transfers) contained SATB2^+^ neurons, demonstrating that they underwent species-appropriate developmental switching to produce upper-layer/late-born cell types during the 10-day culture period. When placed in a macaque environment during this period, human cortical progenitor cells produced deep-layer TBR1^+^ neurons without producing any upper-layer SATB2^+^ cortical neurons (Figures 7D and 7E), continuing to generate the same classes of neurons as they did in their native human environment. Conversely, when we placed macaque cortical progenitor cells into a human environment to ask whether that environment would suppress lineage progression in the macaque, we found that under those conditions, macaque progenitor cells proceeded to switch to generate upper-layer SATB2^+^ neurons, whereas the surrounding human host progenitor cells continued to generate TBR1^+^ deep-layer neurons (Figure 7E).

We further tested the extent to which lineage progression was resistant to environmental cues by coculturing macaque progenitor cells (1:100 dilution) for 30 days with human or macaque progenitor cells from day 25, at which stage both species were initiating production of deep-layer TBR1^+^ neurons (Figure 7F). Clonal assignment during longer-term culture was not possible, due to the very large size of the clones generated. However, we could qualitatively assess whether the donor, GFP-expressing cells underwent lineage progression by analyzing whether they produced SATB2^+^ upper-layer neurons. We found that day 25 macaque progenitor cells generated equivalent numbers of SATB2^+^ neurons over the 30-day period, whether in a human or a macaque environment. Together, these data indicate that lineage progression and cell-type specification in each species are controlled by a cell-autonomous mechanism, resistant to environmental cues.

## Discussion

Using two-dimensional (2D) adherent and 3D organoid stem cell systems, we have found that a major determinant of cerebral cortex size in primates is a species-specific program that controls the output of cortical progenitor cells. This program includes a developmental phase in primates that is not prominent in rodents, during which the progenitor cell population is expanding while also generating deep-layer, early-born neurons. Most striking is the finding that humans, who have notably larger cerebral cortices that contain more neurons than macaques, have a much longer period during which they balance progenitor cell expansion with neurogenesis. This phase enables the production of larger clones from each founder neuroepithelial cell. The proliferative behaviors of human and macaque cortical progenitor cells, outputting as clone size, and the timing of genesis of different classes of cortical neurons were unaffected by exposure to a different species environment in vitro. These data indicate that control of neuronal number and brain size are coordinated in part by a cell-autonomous mechanism that is likely to be under genetic control.

Using a range of approaches based on in vitro differentiation of PSCs from humans, chimpanzee, and two species of macaque in two different cell-culture systems, an adherent 2D system (Shi et al., 2012a) and a 3D organoid system (Kadoshima et al., 2013), we have established that species-appropriate timing of major developmental events in cortical development is maintained in vitro. These events include the generation of all known cortical progenitor cell types, including oRGCs, from neuroepithelial cells (Florio and Huttner, 2014), the temporal order of genesis of projection neurons (Qian et al., 1998), the species-appropriate timing of production of different projection neuron types (Workman et al., 2013), and the maturation of the neuronal electrical properties (McCormick and Prince, 1987). These systems allowed us to carry out a series of investigations into the differences in progenitor cell behaviors between human and other primates during cortical development.

Lineage analysis of primate cortical progenitor cells and computational modeling of neurogenesis revealed that primate cortical progenitor cells go through an extended period during which neurogenesis is balanced with expansion of the proliferating progenitor cell population. During rodent corticogenesis, a small proportion of RGCs increase progenitor numbers during neurogenesis (Noctor et al., 2004). However, we found that the length and extent of the progenitor expansion period in primates were markedly longer compared to rodents and differed between humans and macaques: this phase occurred over approximately 30 days in human compared with 15 days in macaque. We experimentally validated the difference in progenitor cell proliferative behaviors between human and macaque at the population and clonal level, including time-lapse imaging of clonal development over 7 days. The consequence of this feature of human cortical development is to increase overall clone size and thus the total number of cortical neurons, which, ultimately, would increase cortical size in vivo.

Previous models for the increased size of the human cortex, compared with other primates, have proposed two contributing mechanisms. The radial unit hypothesis for cortical development, when applied to the question of cortical expansion, posits that the increase in human cortical size is underpinned by an increase in the founder population of neuroepithelial progenitor cells, without major differences in clone size or number of neurons in each radial unit (Geschwind and Rakic, 2013, Rakic, 2000). Alternatively, the increase in cortical size has been proposed to be a result of an increase in the relative numbers of oRGCs generated later in development, increasing the numbers of later-born, upper-layer neurons (Florio and Huttner, 2014, Geschwind and Rakic, 2013).

The extended period of progenitor cell proliferation during the generation of deep-layer neurons reported here constitutes another mechanism for increasing cortical size, one that operates during the early stages of neurogenesis to regulate clonal size and composition. This additional mechanism increases the clonal output of progenitor cells in humans, compared with macaque, and would result in a disproportionate increase in the size of human cortex if it occurred to the same extent in vivo. Interestingly, this prolonged period of progenitor expansion coincides with the appearance of oRGCs in human and macaque cortex in vivo (Fietz et al., 2010, Gertz et al., 2014, Hansen et al., 2010). It is possible that the increase in early progenitor cell proliferation in primates may lead to, and include, an expansion of the oRGC population. We report here that oRGCs are generated during directed differentiation from PSCs of each primate species, as we previously found in human culture systems (Shi et al., 2012a). However, due to the absence of reliable, quantifiable oRGC-specific markers, it is not currently possible to definitively address this question in the stem cell systems used here.

The observed differences in progenitor cell output and lineage progression between humans and macaques are largely independent of environmental signals in the stem cell systems used here, and they are most likely regulated by a cell-autonomous program. This finding is consistent with previous studies of primary mouse cortical progenitor cells in culture (Qian et al., 1998, Qian et al., 2000). Studies of the genetic basis for differences in cortical development among different mammals, including primates, have identified a range of genetic differences, including single-nucleotide and copy-number variants, with differences in the expression and function of copy-number variants contributing to several aspects of cortical development (Charrier et al., 2012, Keeney et al., 2015). Differences among mammals in gene use, for example, the timing and levels of expression of growth factors and receptors such as PDGF (Lui et al., 2014) and Fzd8 (Boyd et al., 2015) during cortical development, have shown that intercellular signaling is an important regulator of cortical size during in utero development. However, differences in intercellular signaling are unlikely to underlie the differences in progenitor cell behavior observed in vitro, given the cell-autonomous nature of those behaviors in interspecies, mixed cultures.

In conclusion, we have found that the increase in cortical neuronal number in humans compared with nonhuman primates, and the subsequent increase in cortical size, is largely determined by differences in cortical progenitor cell outputs. We have identified a feature of primate cortical development whereby cortical progenitor cells expand their population for an extended period during the genesis of deep-layer neurons, balancing expansion of the progenitor cell population with neurogenesis. This phase of cortical development does not appear to be prominent in rodents (Gao et al., 2014). As well as differing between primates and rodents, this aspect of cortical development varies among primates, leading to differences in cortical size between humans and other primates. Given that this mechanism for controlling cortical size is regulated cell autonomously, in vitro stem cell systems of cortical development provide experimental platforms to identify the relevant cellular mechanisms.

## Experimental Procedures

### PSC Culture and Directed Cortical Differentiation

Human PSCs (H9 ESCs, WiCell Research Institute; Edi2 ESCs, from J. Nichols [Shi et al., 2012a]; NDC1.2 iPSCs [Israel et al., 2012]; and NAS6 iPSCs [Devine et al., 2011]), chimpanzee iPSCs (chimp 00818 iPSCs and 01029 iPSCs [Marchetto et al., 2013]), and macaque ESCs (MF1 ESCs, MF12 ESCs, and MN1 ESCs from E. Curnow, Washington National Primate Research Centre) were cultured either with mitomycin-treated mouse embryonic fibroblasts (MEFs) or under feeder-free conditions in Essential 8 Medium on Geltrex-coated tissue culture plates (Life Technologies). Neural induction was performed as previously described (Shi et al., 2012a, Shi et al., 2012b). Following 12 days of induction, the neuroepithelial sheet was broken up using Dispase (Life Technologies), plated onto laminin-coated plates, and cultured in N2B27-supplemented medium, including 20 ng/mL FGF2 (Peprotech), for 4 days. After day 16 of the induction, cells were maintained in N2B27 medium up to 80 days.

### RT-PCR, Immunofluorescence, and Imaging

Total RNA from cortical cultures was isolated using Trizol (Sigma) and reverse transcribed to cDNA using random hexamer primers (Applied Biosciences). Semiquantitative RT-PCR was performed using primers against *FOXG1*, *NKX2.1*, *DLX1*, *ISL1*, *CTIP2*, *RORB*, *KCNIP2*, *MDGA1*, and *RPS17* and visualized using a Gel Doc XR+ Imager (Biorad). For immunocytochemistry, cells were fixed with 4% paraformaldehyde in PBS and processed for immunofluorescence staining. Primary antibodies used were α-PAX6 (Covance PRB-278P), α-Vimentin (Abcam ab8973), α-phospho-histone H3 (Abcam ab10543), α-atypical PKC (Santa Cruz sc-216), α-Ki67 (BD 550609), α-TBR2 (Abcam ab23345), α-TBR1 (Abcam ab31940), α-MAP2 (Abcam ab10588), α-GFP (Abcam ab4674), α-SATB2 (Abcam ab51502), and α-βIII tubulin (Covance PRB-435P). Immunostained samples were imaged using an Olympus FV1000 inverted confocal microscope.

### Electrophysiology

For electrophysiological recordings, cortical neurons were incubated with artificial cerebral spinal fluid containing 125 mM NaCl, 25 mM NaHCO_3_, 1.25 mM NaH_2_PO_4_, 3 mM KCl, 2 mM CaCl_2_, 25 mM glucose, and 3 mM pyruvic acid and bubbled with 95% O_2_ and 5% CO_2_. Borosilicate glass electrodes with resistance of 6–10 MΩ were filled with an artificial intracellular solution containing 135 mM potassium gluconate, 7 mM NaCl, 10 mM HEPES, 2 mM Na_2_ATP, 0.3 mM Na_2_GTP, and 2 mM MgCl_2_, and positioned over a cortical neuron to form a whole-cell patch. Recordings were made using a Multiclamp 700A amplifier (Molecular Devices), and signals were sampled and filtered at 20 kHz and 6 kHz, respectively. A low-pass Gaussian filter was applied to filter out high-frequency noise.

### Cortical Organoid Generation

Cortical organoids were generated as described (Kadoshima et al., 2013). Briefly, human and nonhuman primate PSCs were dissociated with Accutase (Innovative Cell Technologies), and 12,000 cells were seeded into each well of low-adhesion 96-well plates (Sumitomo Bakelite) in cortical differentiation medium (Glasgow MEM, 20% knockout serum replacement, 100 μM nonessential amino acid, 100 μM sodium pyruvate, 100 μM β-mercaptoethanol, 100 U/mL penicillin-streptomycin, 3 μM IWR1e (Millipore), and 5 μM SB431542). After 18 days, organoids were transferred to a nonadhesive 9-cm petri dish and cultured with postaggregation medium containing DMEM/F12, N2, chemically defined lipid concentrate (Life Technologies), 0.25 mg/ml fungizone (Life Technologies), and 100 U/mL penicillin-streptomycin. As the organoids were cultured for longer periods of time, further supplements were added to the postaggregation medium, including 5 μg/mL heparin (StemCell Technologies), fetal bovine serum (HyClone), 1% growth-factor-reduced Matrigel (BD Biosciences), and B27.

### Clonal Lineage Analysis and Interspecies Mixed Culture Assays

Third-generation replication-incompetent lentivirus was produced by calcium phosphate transfection of HEK293T cells, using pBOP-GFP plasmids combined with packaging plasmids pRSV-Rev, pMDLg/pRRE, and pMD2.G. For clonal lineage analysis, cortical progenitor cells were plated 3 days before retroviral labeling (at incubation day 20, 30, and 40) at a density of 1.0 × 10^5^ cell/cm^3^ and infected with the lentivirus at low titers. Cortical cultures were then “chased” for 2, 6, and 10 days, before being fixed and immunostained. For the in vitro interspecies mixed culture assays, donor progenitor cells were labeled with high-titer lentivirus at day 25 postinduction in two rounds of infection separated by 24 hr. At day 35, donor and host cultures were dissociated and mixed in a 1:100 or 1:1,000 ratio. Cells were plated at a density of 1.0 × 10^5^ cell/cm^3^ and incubated for a further 2, 6, and 10 days.

### Computational Model of Cortical Progenitor Cell Neurogenesis

See Supplemental Experimental Procedures for details.

### Cell-Cycle-Length Measurement and BrdU/EdU Double Labeling

For measuring cell-cycle length, 1 μM EdU was added to the culture medium at day 32. After 2, 8, 14, 20, 26, 32, 38, 44, and 50 further hours in culture, cells were fixed with 4% paraformaldehyde/PBS, and EdU incorporation was visualized using the Click-iT imaging kit (Life Technologies). Cell-cycle lengths were calculated from cumulative labeling as described (Nowakowski et al., 1989). For BrdU/EdU double labeling, human and macaque cortical progenitor cells/neurons were incubated with 1 μg/ml BrdU from day 40. At day 45, BrdU was replaced with 5 μM EdU, and cells were further cultured for 24 hr. At the end of the EdU labeling period, cells were fixed and stained first for EdU and then for BrdU using α-BrdU Alexa Flour 488 antibody (MoBU-1; Life Technologies). Immunostained cells were analyzed by flow cytometry (DakoCytomation Cyan ADP MLE Analyzer, Beckman Coulter).

### Time-Lapse Imaging of Cortical Progenitor Cells

Replication-incompetent retrovirus was used to label neural progenitors at day 35 post-cortical induction. Following incubation for 72 hr, GFP-labeled neural progenitors were imaged every 12 hr for the following 168 hr. N2B27-supplemented neural culturing medium was replaced with Tyrode’s solution containing low potassium and 2 mM CaCl_2_ for imaging (Barreto-Chang and Dolmetsch, 2009).

## Author Contributions

T.O. and F.J.L. designed the study, T.O. conducted the experiments, B.D.S. carried out the lineage analysis and computational modeling, M.C.M. and F.H.G. generated chimpanzee iPSCs, and all authors wrote the paper.

## Supplemental Information

Document S1. Supplemental Experimental Procedures and Figures S1–S4

Document S2. Article plus Supplemental Information
