# Supplementary material for: 2D and 3D Stem Cell Models of Primate Cortical Development Identify Species-Specific Differences in Progenitor Behavior Contributing to Brain Size
Source: Cell Stem Cell. 2016 Apr 7;18(4):467–80. doi: 10.1016/j.stem.2016.03.003 (PMC4826446; doi:10.1016/j.stem.2016.03.003)

Cell Stem Cell, Volume *18*

Supplemental Information

2D and 3D Stem Cell Models of Primate Cortical Development Identify Species-Specific Differences in Progenitor Behavior Contributing to Brain Size

Tomoki Otani, Maria C. Marchetto, Fred H. Gage, Benjamin D. Simons, and Frederick J. Livesey

SUPPLEMENTAL DATA

Supplementary Experimental Procedure 1 – Clonal definition

Supplementary Experimental Procedure 2 – Computational Modeling

Supplementary Figure 1 (Associated with Figure 1): *In vitro* differentiation of ventricular and outer radial glia

Supplementary Figure 2 (Associated with Figure 4): Experimental and computational validation of clonal lineage analysis

Supplementary Figure 3 (Associated with Figure 5): *In vitro* rate of apoptosis and further computational analysis of clonal lineage data

Supplementary Figure 4 (Associated with Figure 6): Cell cycle length measurement in human and macaque cortical progenitor cells

SUPPLEMENTARY EXPERIMENTAL PROCEDURE 1 - CLONAL DEFINITION

Induction frequency and the clonality of labeling assay

The clonal lineage analysis involves the sporadic labeling of cells using a lentiviral reporter construct. By the random nature of labelling, and the potential for cell migration and dispersion, individual clones can become merged leading to a mis-assignment of clonal identity. To assess the frequency of such merger events, we began by scoring the spatial coordinates of labeled cells on multiple plates following a 10 day chase period after clonal labeling of progenitors at day 38 post-cortical induction. We then constructed the nearest-neighbor distribution of marked cells, $g(r)dr$, defined as the probability of that neighboring labeled cells are separated by a distance between r and $r + dr$, (Supplementary Fig. 2B). From this, we could infer that some 90% of labeled cells lie within a distance of 200 μm from another labeled cell. We reasoned that the 10% that lay beyond this distance were post-mitotic at the point of labelling, while the 90% were associated with proximate cells belonging to the same clone.

Based on this assessment, we then assigned clonal identity by grouping labeled cells that lay with 200 μm of another labeled cell (Supplementary Fig. 2C). With this assignment, we identified some $N = 72$ putative clones (with 20 single-cell clones) from 12 plates of area $A = 2500\ x\ 2500\mu$m. If the clonal induction process occurs randomly at a density, $\rho = \frac{N}{A}$, the chance that a labeled cell lies with a distance D of another labeled on induction (and is therefore susceptible to clonal merger) is given by

$$1 - \exp(-\pi\rho D^2).$$

Taking D=200 μm, from the density of multi-cellular clones, we estimated that some 8% (1 in 14) of clones are likely to have been wrongly assigned due to merger events. This level of mis-assignment would not affect the conclusions of our study.

SUPPLEMENTARY EXPERIMENTAL PROCEDURE 2 - COMPUTATIONAL MODELING

Computational modeling of primate cerebral cortex neurogenesis

In the following, we detail the basis of the modeling scheme used to address the clonal data. Our analysis is based on the findings of a recent *in vivo* genetic labeling study of cortical neurogenesis in mouse, which showed that cortical progenitor cells transit sequentially through a symmetrical proliferative phase to a neurogenic phase in which cells make a sequence of asymmetric cell divisions giving rise to intermediate progenitor cells (IP), the latter having variable but limited proliferative potential (*1*). If this behavior were recapitulated in culture, labeled IPs would give rise to small, terminally differentiated clones of maturing neurons. By contrast, progenitor cells labeled in their proliferative phase would give rise to larger clones that expand exponentially, in which the majority of cells remain undifferentiated. Progenitor cells labeled in their neurogenic phase would give rise to a more restricted (linear) growth characteristic, progressively giving rise to IPs that go on to differentiate. On this background, we turn now to the quantitative clonal data to search for evidence of the same general dynamics.

Macaque: Consistent with a progressive shift towards neurogenesis, the clonal data showed a gradual decrease in the proliferative potential of progenitor cells from cultures marked at d20 to those marked at d40 (Fig. 4). From d20, the average clone size increased super-linearly over the 10 day chase, rising to 14±2 (mean±s.e.m) cells per clone, while at d40 the rise is approximately linear to only 6±1 cells per clone. This

reduction in proliferative potential was accompanied by an increase in the frequency of differentiated cells, with some 56±5% of marked cells Ki67- at d40+10 days, compared with just 12±4% at d20+10 days.

The linearity of the increase in average clone size at d40 is suggestive of progenitor cells making asymmetric divisions, as expected for cells already entered into neurogenesis. Therefore, to address the clonal data, we introduced a simple paradigm whose consistency was checked through its ability to predict further aspects of the data. In-line with *in vivo* studies in mouse (*1*), we proposed that the d40 culture comprises a single population of progenitor cells that make a sequence of asymmetric cell divisions, giving rise to IPs. Following transfection, both progenitor cells and IPs are marked in proportion to their frequency in the culture. Following induction, IPs undergo a limited number of rounds of division before terminally differentiating. By contrast, cortical progenitor cells undergo serial rounds of asymmetrical division, giving rise to IPs with "defined" neurogenic capacity. Cell loss is considered to be negligible.

To assess the neurogenic potential of IPs, we first focused on the size distribution of exited clones, defined as those that have fully terminal differentiated over the 10 day chase. From the data it was evident that the time-evolution of the size distribution of exited clones is quantitatively similar for all three ages of culture (d20, d30 and d40), consistent with the capacity of IPs remaining roughly constant over the developmental time course. In particular, referring to the data at d40+10 days (Supplementary Fig. 3C), the peak of the size distribution is biased towards smaller clone sizes, falling to zero at

around 9 cells. Such behavior is consistent with IPs having a maximum neurogenic capacity of around 6-8 neurons, some 2 to 3 times larger than that found from *in vivo* lineage tracing studies in mouse, with smaller clones reflecting the output of IP cell progeny that are marked deeper into their lineage and closer to terminal division. Notably, the clone size distribution also exhibits a striking parity effect where the frequencies of exited clones with an even number of cells are consistently larger than those of odd size.

To capture the approximate form of the exited clone size distribution and parity effect, we introduced a simple model that recapitulates both the average clone size and the shape of the distribution. We proposed that IPs form an equipotent population that either asymmetrically divide (with probability p), or symmetrically differentiate (with probability $1-p$). To capture the parity effect, differentiating progeny may, with probability q, undergo one further round of terminal division. With p=0.37 and q=0.82, the fit of the model to measured clone size distribution (Supplementary Fig. 3C) provides a remarkably faithful parameterization of the data. However, we note that this model represents only a caricature, aimed at capturing the observed size dependence.

Using this "modular" IP cell output, we then addressed the size distribution of the remaining clones that retain at least one Ki67+ cell at 10 days post-labeling (termed persisting), and are therefore likely to be anchored in the cortical progenitor population. Specifically, once entered into neurogenesis, we supposed that cortical progenitor cells undergo a sequence of asymmetric cell divisions at a constant rate λ_{RG}, giving rise to IPs

that divide at rate λ_{IP}. With λ_{IP} estimated from short-term BrdU incorporation at around once per 2 days (Supplementary Fig 4), taking all progenitor cells to be within their neurogenic phase, a fit of the model to the average clone size at the 10 day time point (Fig. 5) gave a cell division rate λ_{RG} of around once per 4 days, slower than IPs. Significantly, with this rate, we found that the model could accurately predict the full size distribution of persisting clones at chase times of 6 (not shown) and 10 days post-transfection (Fig. 5D).

With the analysis of persisting clones complete, we then challenged the model by looking for consistency with the full range of clonal fate data. With the rules above, a stochastic simulation of the model shows that, at "steady state", asymmetrically dividing cortical progenitor cells would constitute some 40% of dividing cells with the remainder IPs. Taking the relative induction frequencies of progenitor cells and IPs to be set in the same proportions, we found that the model could faithfully predict both the total average clone size dependence of the d40 data, as well as the detailed clone size distribution at all three time points (Fig. 5). Significantly, dissecting out the proliferative cell content of clones, we found that the model provided an independent prediction of the progenitor clone size distribution (Supplementary Fig. 3D), including an accurate estimate of the exited clone fraction. As a further check on the predictive capacity of the model, we used the stochastic simulation to estimate the frequency of double labeled cells following 5 days of continuous BrdU incorporation followed by a 24hrs EdU pulse. The model prediction of 29% double-labeled cells compared very favorably to measurements, which show some 31.2%.

Finally, before turning to the human data, we comment qualitatively on the clonal data from the d20 and d30 cultures. While the d30 data is quantitatively similar to d40, a departure of the model for large clone sizes, and a decrease in the fraction of exited clones at the 10 day chase time (38±5% at d30 vs. 56±5% at d40) suggests that some of the cortical progenitor cells labeled at d30 may have yet to enter into neurogenesis. Indeed, for the d20 culture, where only 12±4% of clones terminally differentiate over the 10 day time course, the tail of larger clones becomes much more pronounced (Fig. 4).

Human: As with macaque, the frequency of exited clones in human cultures again suggests a progressive shift towards neurogenesis from d20 to d40. However, even at d40+10, only around 18±4% of clones at 10 days have lost all Ki67+ cells, suggesting that relatively few progenitor cells have entered neurogenesis. Consistent with this hypothesis, the largest clones at 10 days post-labeling are rich in Ki67+ cells, and the average clone size shows a super-linear expansion over the 10-day chase, suggestive of serial rounds of proliferative division (Fig. 4). Of those few clones that have undergone terminal differentiation in the d40 day cultures, their size distribution mirrors that of macaque suggesting that human IPs have a roughly similar neurogenic capacity (Supplementary Fig. 3C).

With the induction of multiple progenitor types at different stages of differentiation, and the potential transfer of cells into neurogenesis during the 10-day chase, an unambiguous deconstruction of static lineage tracing data is infeasible. Therefore, beyond the

qualitative observation of the relative retardation of neurogenesis in human cultures compared to macaque, we looked only for consistency with the modeling scheme. Noting that measurements of proliferation kinetics indicate a similar cell cycle rate (main text), we made the “minimal” assumption that the dynamics of IPs mirror that of macaque (i.e. with the same kinetic and fate parameters as defined above). While there may indeed be important differences in the proliferative and fate potential of IPs between these two systems, providing progenitor cells follow the same pattern of asymmetric division, the model still provides a useful parameterization of the data.

Based on this paradigm, when scaled against the macaque data, the terminal differentiation of 18% of labeled progenitor cells over the d40+10 day time course suggests that some (100-56) x 18/56=14% of marked progenitor cells belong to the compartment of cortical progenitor cells that have already entered into neurogenesis. We then conjectured that the remaining 70% of marked cells belong to the pool of symmetrically dividing cortical progenitor cells. Adjusting the division rate of cortical progenitor cells to the slightly higher value of once per 3 days, we are able to recapitulate the general super-linear rise of the average clone size (Fig. 5). Further, with this parameter, we are able to predict the general structure of the clone size distribution (Fig. 5C). The small systematic departure of the model at the smallest and largest clone sizes may be associated with synchrony in cell cycle progression, which is beyond the resolution of the simplified scheme.

Finally, as a consistency check, we used the model to predict the frequency at which progenitor cells re-enter into cycle in the 24hrs following 5 days of continuous BrdU incorporation. The model prediction of 44% compares favorably with the figure of 48.2% found experimentally.

REFERENCE

1. P. Gao *et al.*, Deterministic progenitor behavior and unitary production of neurons in the neocortex. *Cell* **159**, 775–788 (2014).

Supplementary Figure 1 (Associated with Figure 1): *In vitro* differentiation of ventricular and outer radial glia

A. Immunofluorescence images of $PAX6^+$ RGPs, labeled by constitutive expression of cytoplasmic GFP delivered by lentivirus, revealing morphological characteristics of ventricular RGP and outer RGPs in cortical rosettes of each species. Scale bars, 10μm.

B. Static images from live imaging of a ventricular radial glial progenitor (RGP)-like cell, labeled with cytoplasmic GFP delivered by replication-incompetent lentiviral infection. A progenitor cell (yellow arrowhead) was followed every 30 minutes, and observed to undergo an interkinetic nuclear migration (IKNM)-like movement prior to cell division (red and blue arrowheads indicate two daughter cells). Scale bar, 5μm.

C. Images of an outer RGP-like cell, taken every 30 minutes. The oRGP-like cell translocated its cell body basally before the cell division by mitotic somal translocation.

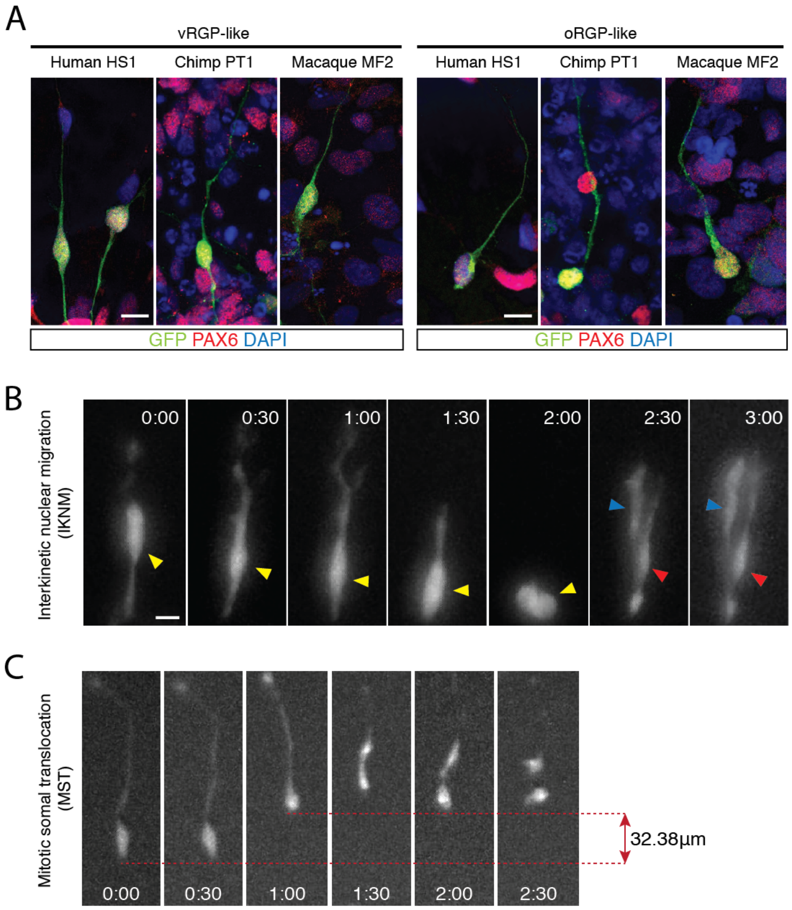

Supplementary Figure 2 (Associated with Figure 4): Experimental and computational validation of clonal lineage analysis

- **A.** Representative immunofluorescence image showing distribution of macaque MF2 clones as used for clonal lineage analysis. MF2 cortical progenitors were labeled with replication incompetent lentivirus, expressing either GFP or mCherry, at d38 and analysed 10 days later (d38+10). The histogram shows the proportion of clones that had a mixture of GFP and mCherry-positive cells. Scale bars, 500µm. Error bars, s.d.
- **B.** Nearest-neighbor probability distribution obtained from measurements of cells marked by lentiviral labeling from a total of n=14 culture plates following a 10 day labeling period (d38+10). The distribution shows a steep drop and shoulder at D=200 μm (arrow). Neighboring labeled cells with a larger separation are associated with the marking of multiple cells, while those with a shorter separation are presumed to belong to the same single clone.
- **C.** Spatial coordinates of cells in clones derived from 12 plates obtained by designating labeled cells separated by a distance of D=200 μm or less as belonging to the same clone. Different clones are marked in different colors.
- **D.** Box plots comparing clonal lineage data for multiple cell lines for human and macaque (each dataset represents a genetically distinct line) 10 days after clonal labeling of progenitors at d30 or 40. Each box represents 50% of the data (from lower to upper quartiles of the data), the red square inside depicts the mean, and the line within each box represents median values. Whiskers extend from the box to the lowest and highest data points that are still within a 1.5-interquartile range of the lower and upper quartiles. Hollow circles represent outliers.

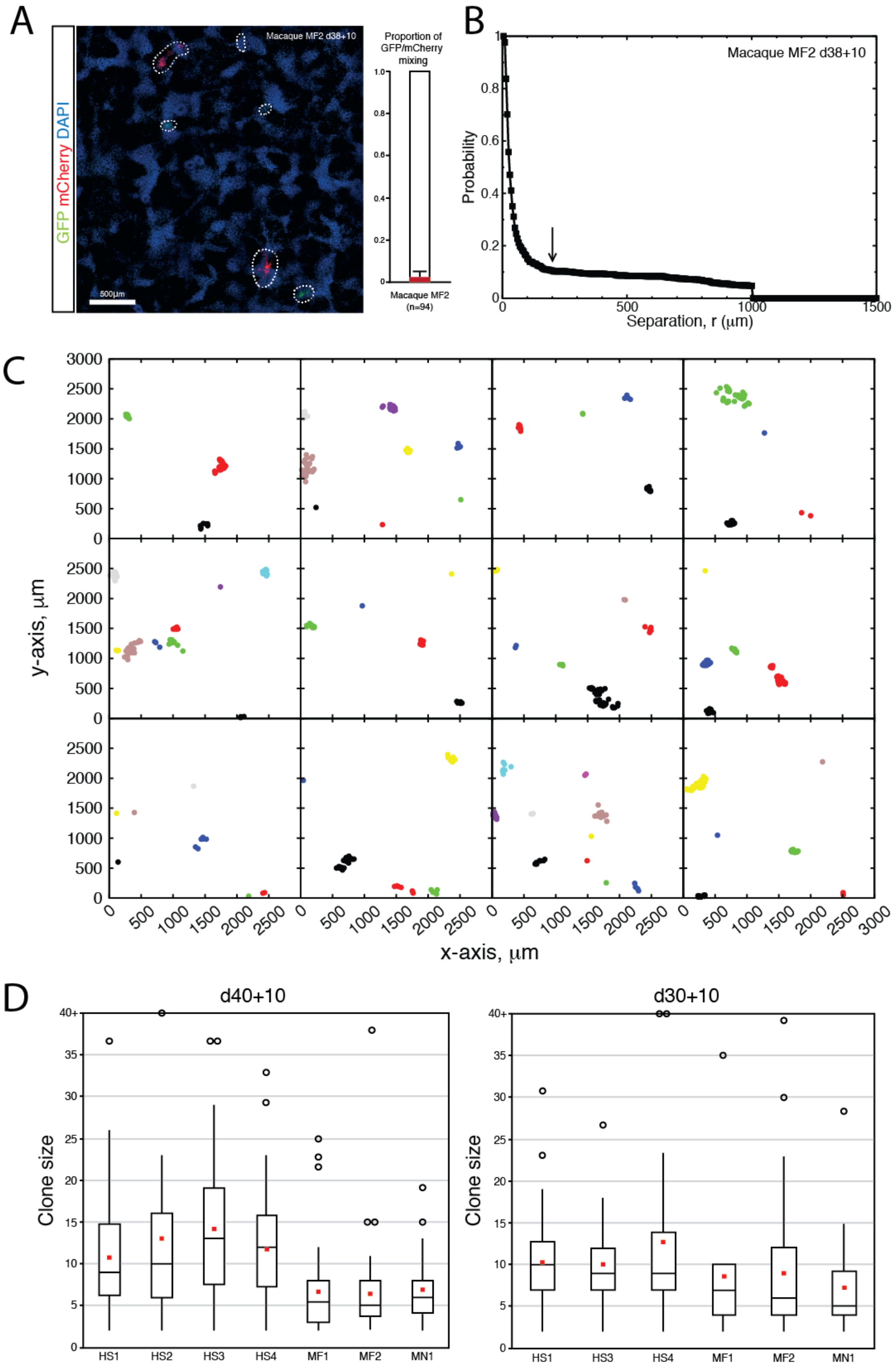

Supplementary Figure 2

Supplementary Figure 3 (Associated with Figure 5): In vitro rate of apoptosis and further computational analysis of clonal lineage data

A. Images from live imaging of a cortical neuron undergoing apoptosis (yellow arrowhead). Scale bar, 50µm.

B. Histogram showing the proportion of apoptotic cells observed in culture. There is no difference in the rate of apoptosis between different species. Error bars, s.d.

C. Clone size distributions of human and macaque exited clones following a 10 day-chase period after clonal labeling of progenitors at day 40 (d40+10). Blue squares represent theoretically predicted values.

D. Histogram of measured and theoretical values of the distribution of Ki67+ progenitor cell content in macaque clones at the d40+10 time point. (See supplementary notes on computational model for details). Error bars, s.d.

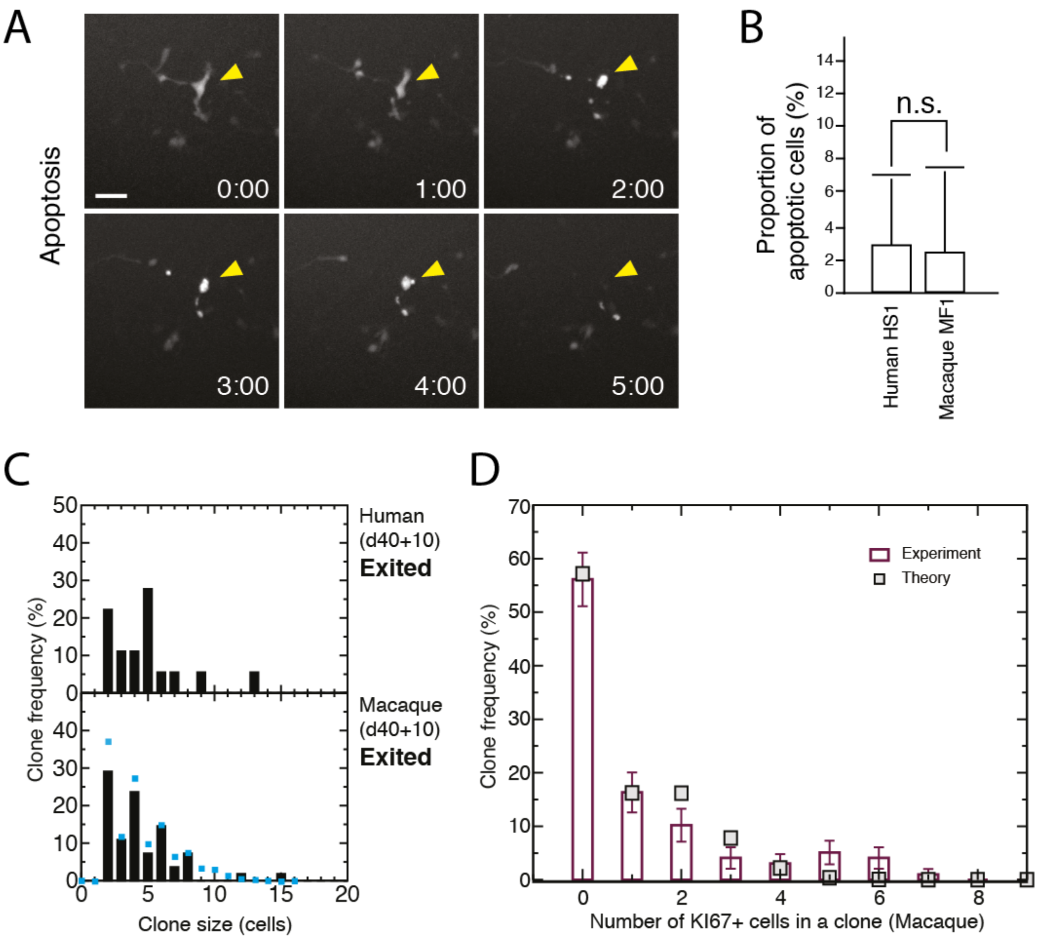

Supplementary Figure 4 (Associated with Figure 6): Cell cycle length measurement in human and macaque cortical progenitor cells

A. Experimental design of the cumulative labeling assay used to measure cell cycle length (see Methods). EdU was added at d32 and kept continuously in the medium for the duration of the experiment. Cells were sampled after different time periods (2, 8, 14, 20, 26, 32, 38, 44 and 50 hours) for immunostaining.

B. Representative immunofluorescence images of cortical cultures following EdU incubation for 2, 8 and 14 hours, immunostained for EdU, Ki67 and PAX6. Scale bar, 100μm.

C, D. Graphs showing the increase in the percentage of human and macaque $PAX6^+Ki67^+$ (C; RGCs) or $PAX6^-Ki67^+$ cells (D; all other progenitor types) that have incorporated EdU over hours of incubation. Cell cycle lengths of $PAX6^+/Ki67^+$ progenitor cells are significantly longer in human compared with macaque ($p = 2.03x10^{-3}$), while there is no difference for $PAX6^-/Ki67^+$ progenitor cells. The solid portion of each bar graph represents S-phase of cell cycle, error bars represent s.d.

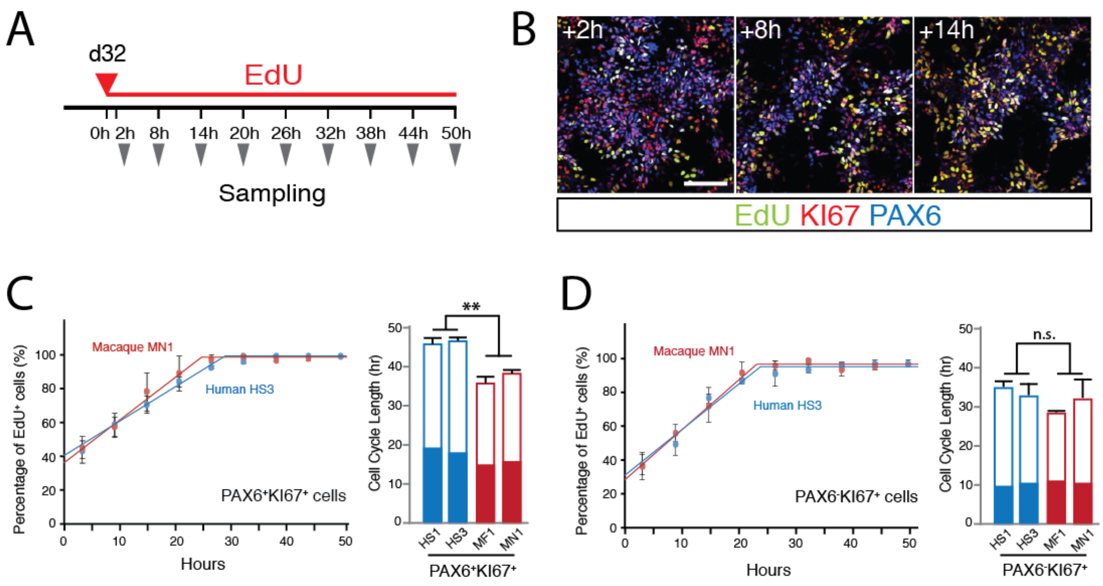

Supplement: Document S1. Supplemental Experimental Procedures and Figures S1–S4 [file mmc1.pdf]
